# Interference of manganese removal by biologically-mediated reductive release of manganese from MnO_x(s)_ coated filtration media

**DOI:** 10.1016/j.wroa.2018.100009

**Published:** 2018-11-13

**Authors:** Lindsay E. Swain, William R. Knocke, Joseph O. Falkinham, Amy Pruden

**Affiliations:** a Via Department of Civil and Environmental Engineering, Virginia Tech, Blacksburg, VA 24061, USA; b Biological Sciences Department, Virginia Tech, Blacksburg, VA 24061, USA

**Keywords:** Drinking water treatment, Manganese removal, Media filtration, Manganese oxides, Manganese-reducing bacteria

## Abstract

Discontinuing application of pre-filter chlorine is a common water treatment plant practice to permit a bioactive filtration process for the removal of soluble Mn. However, soluble Mn desorption has sometimes been observed following cessation of chlorine addition, where filter effluent Mn concentration exceeds the influent Mn concentration. In this paper it is hypothesized that Mn-reducing bacteria present in a biofilm on the filter media may be a factor in this Mn-release phenomenon. The primary objective of this research was to assess the role of Mn-reducing microorganisms in the release of soluble Mn from MnO_x(s)_-coated filter media following interruption of pre-filtration chlorination. Bench-scale filter column studies were inoculated with *Shewanella oneidensis* MR-1 to investigate the impacts of a known Mn-reducing bacterium on release of soluble Mn from MnO_x(s)_ coatings. *In situ* vial assays were developed to gain insight into the impacts of MnO_x(s)_ age on bioavailability to Mn-reducing microorganisms and a quantitative polymerase chain reaction (qPCR) method was developed to quantify gene copies of the *mtrB* gene, which is involved in Mn-reduction. Results demonstrated that microbially-mediated Mn release was possible above a threshold equivalent of 2 × 10^2^*S. oneidensis* MR-1 CFU per gram of MnO_x(s)_ coated media and that those organisms contributed to Mn desorption and release. Further, detectable *mtrB* gene copies were associated with observed Mn desorption. Lastly, MnO_x(s)_ age appeared to play a role in Mn reduction and subsequent release, where MnO_x(s)_ solids of greater age indicated lower bioavailability. These findings can help inform means of preventing soluble Mn release from drinking water treatment plant filters.

## Highlights

• No measureable release of soluble Mn from autoclaved sand coated with MnO_x(s)_.
• Elevated effluent soluble Mn when MnO_x(s)_-coated media inoculated with *S. oneidensis* MR-1.
• Inoculated columns contained more copies of Mn-reduction functional gene *mtrB*.
• Apparent reduction of bioavailability of MnO_x(s)_ media coating with aging.
• Results suggest that biological Mn reduction can release soluble Mn from MnO_x(s)_ coated media.

## Graphical abstract

**Figure:**
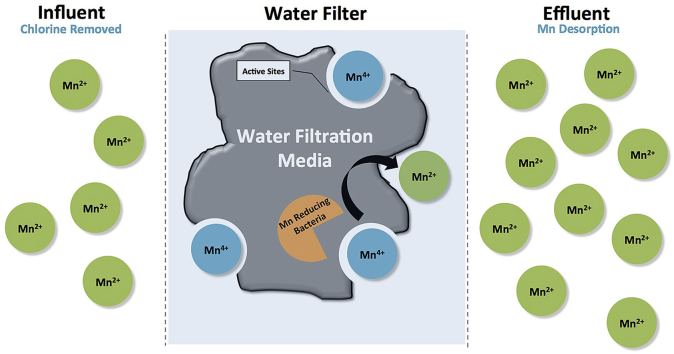

## 1 Introduction

Mn is a challenging drinking water contaminant, owing to its widespread natural occurrence and multiple oxidation states (e.g., Mn(II), Mn(III), Mn(IV) in water). Elevated Mn concentrations (SMCL = 0.05 mg/L in U.S.) can present a significant aesthetic concern as the water travels through the distribution system and into a consumer's home plumbing. Upon entering the consumer's home, water that contains oxidized Mn is often characterized by a blackish-brownish color, which can lead to water discoloration complaints (Sly et al., 1990) and an undesirable metallic taste (Sain et al., 2014).

Microbial metal reduction is central to the geochemical cycling of Fe, Mn and C in redox-stratified waters (Johnson, 2006). Yet, little is known about the microbial metabolic mechanisms by which the Mn reduction process takes place. In contrast to reduction of iron, the current state of understanding Mn-reduction is that it does not provide energy to the cell (Lin et al., 2012). Under neutral pH conditions, Mn oxides are highly insoluble and can be crystalline in nature (Yang et al., 2013). For reduction to occur, microorganisms are required to transfer electrons to Mn oxides that are external to the cell wall or membrane, since contact with a localized inner membrane electron transport chain is not possible (Lovley et al., 2004). It is generally thought that Mn(IV) reduction occurs directly at the outer membrane via single two-electron successive transfers, resulting in Mn(II) as the final product (Thamdrup, 2000). The first electron transfer forms soluble Mn(III) as a temporary intermediate before a final electron transfer and reduction to Mn(II). The first electron transfer step increases the bioavailability of Mn by reductive solubilization, while the second step is coupled to the production of inorganic carbon (Lin et al., 2012).

*Shewanella* has been used as a model metal-reducing bacterium due to its ability to utilize a wide variety of electron acceptors and its capacity to transfer electrons to solid metal oxide compounds (Osterman et al., 2008). The electron transport chain of *S. oneidensis* is made up of several different interacting proteins, which allow the utilization of extracellular electron acceptors for Mn reduction (Szeinbaum et al., 2014). The main outer membrane protein complex is MtrCBA, an electron channel that allows their passage to extracellular metal hydroxide complexes (Burnes, 2000). MtrCBA is mainly associated with the reduction of Mn(IV). An outer membrane β-barrel protein, MtrB, allows interaction and electron transfer to extracellular metal hydroxide complexes and has been shown to be required for Mn(IV) and Mn(III) reduction (Lin et al., 2012).

One of the more common methods of soluble Mn removal in water treatment facilities is through adsorption onto MnO_x_(s)-coated filtration media (Knocke et al., 1991; Tobiason et al., 2008). This process has been referred to as the natural greensand effect and is a self-regenerating process that continues to remove additional soluble Mn (Brandhuber et al., 2013). In a few water treatment plants (WTPs) where adsorption of soluble Mn to oxide-coated filtration media is the main method utilized for soluble Mn removal, Mn desorption and release from the media has been observed upon cessation of pre-filter free chlorine addition (Islam, 2010). In certain cases, effluent Mn exceeded the influent Mn concentration for extended periods of time (e.g., 1–2 weeks) after chlorine application ceased (Gabelich et al., 2006). This desorption phenomenon has specifically been documented at the Aquarion Water Company (AWC) Stamford WTP (Stamford, CT) (Tobiason et al., 2008) and at the Henry J. Mills WTP (Riverside, CA) (Gabelich et al., 2006). Mn desorption from coated filter media has also been observed in the laboratory using filtration media from the AWC Lantern Hill WTP (Stonington, CT) (Islam, 2010). Mn release appeared to be due to biologically-mediated desorption from the anthracite filtration media. In addition, Islam (2010) indicated that Mn-reducing populations were only capable of growth on filtration media and reduction of Mn in the absence of chlorine. Consistent with the above observations, Cerrato et al. (2010) were able to isolate Mn-reducing bacteria from the media of WTPs operating in Virginia and North Carolina. Notably, some of these bacteria, mainly *Bacillus*, were capable of both oxidizing and reducing Mn. Further, recent studies have revealed that a wide diversity of microbes inhabit WTP media, even under chlorinated conditions (Chiao et al., 2014).

The overall goal of this research was to evaluate the potential for biologically-mediated Mn-reduction from MnO_x_(s)-coated filtration media during drinking water treatment. The specific objectives were to: 1) characterize Mn release from columns with varying extent of MnO_x(s)_ coating on the media under conditions of reduced microbial activity; 2) compare Mn-desorption from laboratory-scale filter columns containing porous media with and without inoculation of *S. oneidensis* MR-1, a known Mn-reducing microorganism; 3) develop and apply a molecular assay for the quantification of the *mtrB* marker gene associated with biological Mn-reduction; and 4) investigate the effects of MnO_x(s)_ age on bioavailability and Mn-reduction capacity. The goal was to generate information to inform improved operation of media filters at WTPs to optimize biological benefits of removing organic carbon via biofiltration while minimizing potential concerns related to Mn release.

## 2 Experimental methods and materials

The following text describes the experimental and analytical methods applicable to this study. More detailed information may be found in Swain (2016).

### 2.1 Laboratory bench-scale filter column experimental and analytical procedures

Bench-scale filter columns were set up to produce Mn breakthrough curves as well as replicate Mn removal and desorption trends observed in full-scale WTPs. Each laboratory bench-scale filter contained a filter media (sand or anthracite), received an influent that contained soluble Mn and aluminum (Al, primarily as Al(OH)_3_(s)), and had free chlorine applied for a defined initial amount of time to promote formation of MnO_x_(s) coating on the media and active site regeneration for the consistent uptake of soluble Mn. Particulate Al(OH)_3_(s) was added at a low concentration (approximately 200 μg/L as Al) to mimic the presence of carryover floc from sedimentation basins in “real-world” surface WTPs (Jones, 2012). During each study, free chlorine application ceased after a set amount of time and the column influent and effluent were measured to evaluate the amount of soluble Mn passing through the column media.

Glass columns were 24 inches in height and had a 7/16-inch inner diameter. Plastic tubing was employed to transfer feed solutions to each column and transport filtered effluent away from the column. Six inches of media depth was used in each column experiment.

Influent feed solutions were prepared with deionized water in five-gallon increments and stored in larger plastic reservoirs. Each column had two influent feed solutions that were mixed just prior to entering the filter column. The primary feed solution contained dissolved Mn, alkalinity, dissolved and particulate Al. The second feed solution always contained alkalinity and contained free chlorine during the time period when an experiment required the presence of free chlorine in the filter-applied water. Once mixed together the resulting influent feed solution characteristics of the water for each experiment were achieved.

Columns were backwashed as needed when excessive head loss occurred or at least once daily. Backwashing was performed by pumping 1 L of deionized water in an upflow direction through the column at a rate of 25 gpm/ft^2^. This flow rate corresponded to an approximate 30% media expansion.

There were two distinct sets of filter column experiments on two different MnO_x_(s)-coated filter medias to assess the potential role of Mn-reducing bacteria on Mn release. Those procedures are provided in the following text.

#### 2.1.1 Abiotic studies for assessing potential soluble Mn release from media

An initial set of experiments were designed to assess the potential for soluble Mn release from filter media under abiotic conditions. These served as a “control” to determine whether soluble Mn release would occur from MnO_x_(s)-coated media purely by chemical means (e.g., cessation of pre-filter chlorine addition). These experiments employed the use of new FilterSil sand (uniformity coefficient 2.65; effective size 0.5 mm), which initially had no MnO_x_(s) coating present. The media was autoclaved prior to the start of MnOx(s) coating and laboratory experiments to minimize microbial activity. An initial MnO_x_(s) coating was generated on the sand media through application of soluble Mn (200 μg/L) and free chlorine (1–2 mg/L) for a defined period (either 5 or 15 days). Extraction of the media coating by hydroxylamine sulfate addition indicated an MnO_x_(s) coating level of 1.5 mg Mn/g media and 4.9 mg Mn/g media after 5 and 15 days of free chlorine application respectively. This insured the presence of ample MnO_x_(s) coating for evaluating the potential for Mn reduction and release after free chlorine addition ceased in these experiments. Additional influent water characteristics to the sand media were: pH – 7.0 to 7.3, alkalinity - 2 meq/L, total Al – 200 μg/L, and TOC - <0.5 mg/L. Influent feed solutions were pumped into the column to yield a total hydraulic loading rate of 4 gpm/ft^2^.

Following the initial period (5 or 15 days) of MnO_x_(s) coating deposition on the sand, the free chlorine was eliminated from the feed water. Column effluent was monitored for the presence of soluble Mn to evaluate the potential for actual release of Mn from the media coating.

#### 2.1.2 Experiments employing Mn-reducing bacteria to assess potential for Mn release from media coatings

A second set of experiments sought to assess the potential role of Mn-reducing bacteria in promoting Mn release from MnO_x_(s)-coated media. These experiments employed anthracite coal media (uniformity coefficient 1.4; effective size 0.9–1.0 mm) obtained from the Harwood Mills Waterworks WTP (Newport News, VA). The Harwoods Mill media was chosen for use due to its significant (28–30 mg Mn/g media) MnO_x_(s) coating that was present. This WTP employs free chlorine addition to the filter-applied water for soluble Mn control.

The influent characteristics for the anthracite media experiment were as follows: pH – 6.3 to 6.6, alkalinity 2 meq/L, soluble Mn – 50 μg/L, and total Al – 200 μg/L. The slightly lower pH condition employed (in relation to the abiotic study) was due to the fact that the typical filter-applied pH at the Harwoods Mill WTP is between 6.0 and 6.5. As such, it was desired to use a pH application similar to what the media experienced in the WTP. A free chlorine concentration of 0.5–1.0 mg/L (again, comparable to Harwoods Mill WTP practice) was present in both columns for the initial five-day period of the test, after which chlorine was no longer fed to the column. Whereas the abiotic study had no TOC added to the filter-applied water, this experiment incorporated a small amount of sodium acetate (influent TOC = 0.5 mg/L as C) to serve as a readily degradable organic substrate.

One of the columns in this study was inoculated with *S. oneidensis* MR-1 (a well-characterized Mn-reducing bacterium) while the other column had no microbes applied to the filter media. *S. oneidensis* MR-1 was obtained courtesy of Dr. Kenneth Nealson from the University of Southern California. To prepare the *S. oneidensis* MR-1 inoculum, a 10-μL sterile loop full of culture from an R2A agar (BD Difco) plate was transferred to 100 mL of R2A broth (BD Difco). The flask was incubated for 48 h in a shaking water bath at 30 °C. *S. oneidensis* MR-1 was then used to inoculate a 1 L volume of R2A broth and again incubated for 48 h in a shaking water bath at 30 °C. The entire volume of culture was further allowed to acclimate to room temperature for an additional 24 h. The culture was centrifuged at 5,000 g for 20 min to concentrate the pellet. The pellet was suspended into a 200 mL volume of modified Mn-reduction broth, which contained per liter of 10 mM HEPES buffer (pH 7.4): 0.2 g yeast extract and 2 g sodium acetate. The modified Mn-reduction broth, with concentrated *S. oneidensis* MR-1 inoculum, was poured over the anthracite media in the column. The culture was allowed to flow through the column for a short time and then was used to fill the column above the media level. The column was left undisturbed for 24 h, after which it was lightly backwashed. Additional culture was added to the column and was again subject to a short duration of flow. Culture filled the column above the media height and was left undisturbed for an additional 24 h. After the period of column inoculation, influent flow was introduced to the filter.

These two columns were monitored for effluent Mn concentrations before and after the cessation of pre-filter chlorine to evaluate whether the presence of the Mn-reducing bacteria potential led to Mn release from the media coatings.

#### 2.1.3 Analytical methods for filter column studies

The pH of the influent feed solutions to the filter columns was monitored and adjusted with concentrated hydrochloric acid (HCl) as needed. A HACH HQ40d pH meter was calibrated daily before measurements were taken. A HACH reagent set (DPD method) was utilized for the measurement of free chlorine. Influent and effluent bench-scale column samples for Mn analysis were preserved in 2% trace metal grade nitric acid and were analyzed using inductively coupled plasma mass spectroscopy (ICP-MS).

A description of the term “soluble Mn” is provided for clarity. The influent solution to any media column contained only dissolved manganous (Mn(II)) ion. Likewise, the relatively brief (less than 2 min) contact time between the dissolved Mn(II) and free chlorine prior to application to the coated media was too short to promote any significant Mn(II) oxidation and formation of particulate MnO_x_(s) in the water applied to the media (Brandhuber et al., 2013). Preliminary testing with 0.45 μm membrane filtration of column effluent samples showed no significant presence of particulate, oxidized MnO_x_(s) being present, which is consistent with practical experience when soluble Mn is the only source in the feed. As such the protocol for experimental testing did not involve 0.45 μm filtration of the column effluent samples and assumed that Mn present in the column effluent was in the soluble form.

### 2.2 Molecular detection of Mn-Reducing microorganisms

#### 2.2.1 Sample collection for molecular analyses

DNA was isolated from standard pure culture suspensions, laboratory samples, and environmental samples using the PowerSoil kit from MoBio Laboratories, Inc. (Solon, OH) for use in quantitative polymerase chain reaction (qPCR). For extraction from anthracite media, 0.25 g of each sample was measured and transferred to each extraction kit vial. For liquid samples and standards, suspensions were mixed and 250 μL was added into the extraction kit vials. Samples and standards were stored at −20 **°**C until extraction.

#### 2.2.2 *S. oneidensis* MR-1 qPCR

The *S. oneidensis* MR-1 16S rRNA gene RT-qPCR methods described by Schicklberger et al. (2011) were modified for qPCR. SsoFast EvaGreen Supermix (Bio-Rad, Hercules, CA) was used, with a reaction volume of 10 μL. The mastermix contained 5 μL of SsoFast EvaGreen supermix, 0.8 μL of the forward and reverse primers, 2.4 μL molecular grade water and 1 μL DNA template. The DNA template was quantified using a Qubit Fluorometer (Bio-Rad) and diluted to a concentration where 5–15 ng/μL was added to each well before use in qPCR. The optimal melting temperature was determined by a temperature gradient and melt curve ranging from 57.0 **°**C to 51.0 **°**C, where 57.0 **°**C was chosen as the optimal melting temperature for this protocol. A standard curve was developed using DNA extracts from suspensions of *S. oneidensis* MR-1 culture of known colony-forming units per mL density to quantify this organism from laboratory samples and bench-scale filter column studies.

#### 2.2.3 mtrB gene detection

Multiple sequence comparison by log-expectation “MUSCLE” alignment (Edgar, 2004) was chosen as the method to aid in design of the *mtrB* primer set. From the NCBI GenBank website, 10 MtrB protein sequences were chosen for gene alignment based off the targeted sequence description (Table S1). The final *mtr*B primers obtained from the alignment is as follows: Forward 5′-CSTTCAACVACATGGCCG-3′ and Reverse 5′-SGAGATCTCSAGCAGGTC-3’.

For a 10-μL qPCR reaction volume, the mastermix contained 5 μL of SsoFast EvaGreen supermix, 0.8 μL of the forward and reverse primers, 2.4 μL of molecular grade water and 1 μL of DNA template. DNA template was added into the reaction wells at a concentration between 5 and 15 ng/μL. Annealing temperature ranges were tested between 50.0 **°**C and 58.0 **°**C, where 50.0 **°**C was chosen as optimal. To validate the assay, strains of *Bacillus* spp. that were verified not to have the capability to reduce Mn were analyzed as a phylogenetically-related negative control. This validation confirmed non-detect levels of the *mtrB* gene for the negative control strains.

### 2.3 In-situ Mn-Reduction vial assay

An *in situ* vial assay was developed to semi-quantitatively evaluate the bioavailability to *S. oneidensis* MR-1 of various aged MnO_x(s)_ samples. Various modifications, detailed in the following sections, were made from the protocol established by Nealson et al., (1991). A total of five MnO_x(s)_ samples were examined using the vial assay developed. Three of the MnO_x_(s) samples were prepared by adding potassium permanganate (KMnO_4_) at a stoichiometric dosage to waters containing soluble Mn (obtained by dissolving manganous chloride (MnCl_2_)). Once the reaction was complete, the MnO_x(s)_ formed was centrifuged at 10,000 g for 5 min to form a pellet and remove excess water. The MnO_x(s)_ samples were washed by resuspension in nanopure water, and re-centrifuged to form a new pellet three times. These solids were precipitated approximately one to seven months prior to use in the vial assay, and were identified according to synthesis date (7-28-15, 11-18-15 and 1-11-16). A fourth MnOx(s) sample (2008) was a purchased MnO_2_(s) oxide from Sigma that had been stored in the laboratory for approximately 7 years. The fifth MnO_x(s)_ sample (7-8-15) was artificially “aged” by drying the formed MnO_x_(s) at 103 **°**C for 24 h. An attempt was made to inactivate any microbes possibly present on the MnO_x_(s) by exposure to an extremely strong free chlorine environment. Chlorine inactivation of the MnO_x(s)_ samples was completed by soaking the samples in a strong chlorine solution for sufficient time to yield a C_t_ product of 10,000 mg/L-min. After the desired C_t_ of 10,000 mg/L-min was reached, the MnO_x(s)_ samples were washed three times using sterile deionized water.

A semisolid manganese reduction agar contained the following per liter of 10 mM HEPES buffer (pH 7.4): 0.2 g yeast extract, 2 g sodium acetate and 3 g agar (0.3%). The media was autoclaved on a 20-min liquid cycle and was allowed to cool before the addition of MnO_x(s)_. To determine the responsive range of the assay, percent transmission at 540 nm was measured for lab-synthesized MnO_x(s)_ solids (1-11-16 and 2008 samples) over the range of 0–1.0 g/L. Percent transmission versus MnO_x(s)_ solids concentration fit a polynomial curve with an R^2^ > 0.95. Based on this, a target initial MnO_x_(s) sample addition of 0.7 or 0.35 g/L was selected in order to maintain the assay within a measurable range over the duration of the assay. As noted above, the commercial MnO_2_(s) solids and the dried (at 103 °C) MnO_x(s)_ solids were much darker in color and thus were added at the lower initial concentration (0.35 g/L) to achieve a comparable initial transmittance measurement of 4–8% across the vial assays. A volume of 10 mL of the Mn-reduction agar, with MnO_x(s)_ added, was placed into glass vials for inoculation with the S. *oneidensis MR-1* bacteria.

Vials were inoculated with fresh *S. oneidensis* MR-1 cultures from R2A agar plates, which were incubated overnight at 30 **°**C. Cultures were removed from the agar plate with a sterile loop and suspended in 1.5 mL of sterile deionized water and subject to serial dilution. A 100-μL aliquot of the desired *S. oneidensis* MR-1 suspension was added into each vial and was vortexed. Each inoculum was enumerated using standard heterotrophic pour plate methods on R2A agar before the addition into vials. A volume of 100 μL of sterile deionized water was added into the negative (uninoculated) control vials.

Percent transmittance of light at 540 nm was used as a comparative surrogate parameter for semi-quantitatively estimating the degree of Mn reduction occurring within the vial. As MnO_x_(s) was reduced to soluble Mn, the percent transmittance of the suspension in the vial increased. Percent light transmittance was measured frequently (a minimum of every 1–2 days) using a spectrophotometer (Coleman Junior, Model 6A, Maywood, IL) that was calibrated at the 540 nm wavelength before each use (Gerhardt et al., 1981). Abiotically-reduced positive control vials were prepared by adding 0.020 g of hydroxylamine sulfate suspended in 100-μL of sterile deionized water was added into a parallel set of vials for each type of MnO_x_(s) sample. This allowed for an estimate of the percent transmittance of a sample wherein essentially 100% of the MnO_x_(s) added was reduced to soluble Mn.

## 3 Results and discussion

### 3.1 Abiotic filter media study - Mn sorption and release from MnO_x_(s)-Coated virgin sand

An initial experiment involved an abiotic study of the potential reduction of MnO_x_(s) media coatings. MnO_x_(s) coatings were deposited onto autoclaved sand by interaction between soluble Mn and free chlorine on the media for an initial period of either 5 or 15 days (the longer time led to greater MnO_x_(s) deposition on the sand). After the prescribed period had elapsed, the free chlorine was removed from the filter-applied water and only soluble Mn loading continued.

**Fig. 1:**
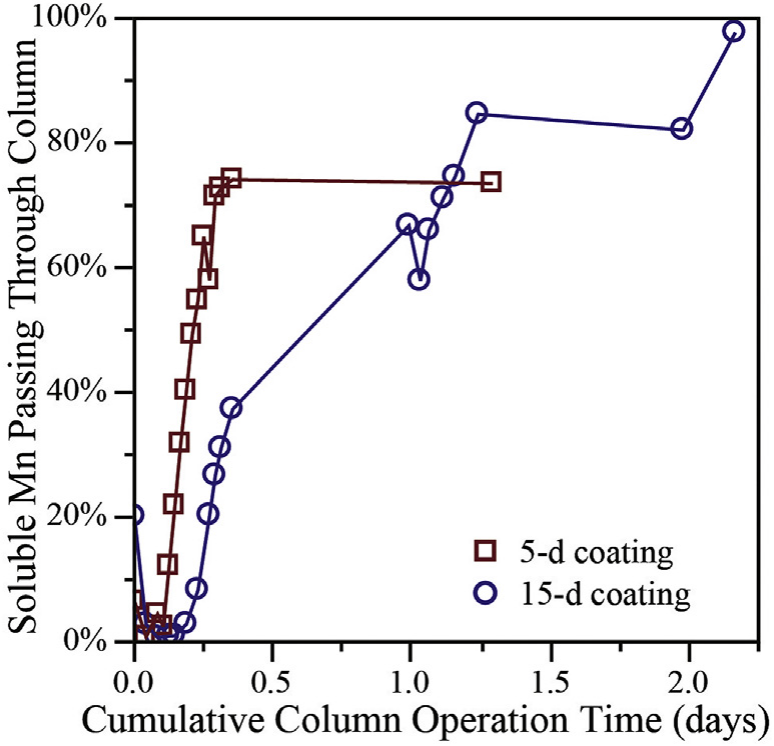
Comparison of the percentage manganese measured in effluent of columns containing sand media coated with MnOx_(s)_ for 5 days versus 15 days, relative to influent feed concentration (0.2 mg/L Mn), after the discontinuation of free chlorine in the feed.

Results from this abiotic study are shown in Fig. 1. Time zero was set as the time chlorine addition ceased for each column. Soluble Mn breakthrough occurred more quickly when the media in the columns had been coated for only five days. The rate of soluble Mn removal is known to be directly associated with surface MnO_x(s)_ concentration, which impacts the amount of available active sites on a filter media (Knocke et al., 1991). Faster breakthrough from the 5-day coated columns would be expected, as the MnO_x_(s) coating level was only 1.5 mg Mn/g media whereas 4.9 mg Mn/g media of MnO_x_(s) coating was found on the 15-day coated media. Knocke et al. (1991) demonstrated that the number of MnO_x(s)_ active sites positively correlate with the degree of MnO_x(s)_ coating. Therefore, the column with the 15-day MnO_x(s)_ coating had increased adsorption capacity on the media surface, which resulted in slower Mn breakthrough.

Importantly, the concentration of Mn passing through the media depth never exceeded influent Mn concentrations for either the 5-day or 15-day coated columns. The breakthrough trends from this column experiment can be explained by the loss of Mn uptake capacity on the media sites due to Mn uptake becoming saturated, given that regeneration with free chlorine was no longer taking place. The cessation of free chlorine to filters in some full-scale WTP (Gabelich et al., 2006) and laboratory studies (Islam, 2010) have shown Mn desorption that results in effluent Mn concentrations exceeding the influent for a period of several hours up to 1–2 weeks, apparently due to reduction of Mn from media coatings and release to the filter effluent. These abiotic column studies did not yield situations where the column effluent Mn concentration exceeded the influent Mn level of 0.2 mg/L. As such, operation of the columns under abiotic conditions did not yield evidence of Mn reduction in the available MnO_x_(s) media coatings, suggesting a lack of purely chemical mechanisms for promoting Mn reduction on the media.

### 3.2 Laboratory bench-scale filter study - Mn release from harwood mills anthracite media with and without inoculum

**Fig. 2:**
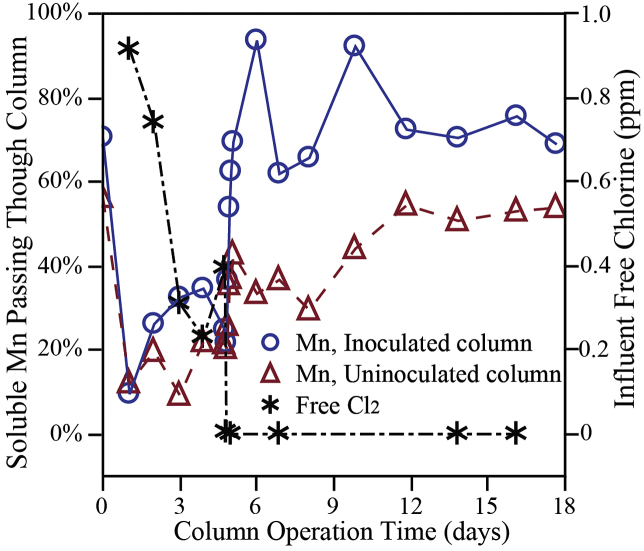
Percentage of Mn measured in effluent of “Inoculated” (*S. oneidensis* MR-1) and “Uninoculated” (sterile inoculum) Columns.

Data related to the passage of soluble Mn through the anthracite media filter depth for both the “Inoculated Column” (*S. oneidensis* MR-1 inoculum) and “Uninoculated Column” are presented in Fig. 2. During the initial five days when free chlorine was applied, the Mn breakthrough in the inoculated column was consistently greater when compared to the uninoculated column. Likewise, after free chlorine addition ceased, the release of soluble Mn was much greater for the inoculated column. Experimental conditions (e.g., pH, applied Mn concentration, applied free chlorine concentration, chlorine demand across the media depth, time of chlorine feed cessation, etc.) between both columns remained the same through the duration of the experiment. As such it is hypothesized that the difference in Mn breakthrough observed could be attributed to the Mn reduction activity of the *S. oneidensis* MR-1 inoculum.

Lovley and Phillips (1988) showed that *S. oneidensis* MR-1 coupled the oxidation of electron donors (similar to the acetate supplied in the feed to these two columns) to Mn reduction. In this specific experiment, acetate was supplied as a readily oxidized substrate that could have been coupled with the reduction of MnO_x(s)_ from the media surface by the Mn-reducing microorganisms. This result compares well to Islam (2010), who were able to demonstrate a relationship between applied acetate concentration to MnO_x(s)_-coated media and subsequent soluble Mn release from that media.

#### 3.2.1 S. oneidensis MR-1 quantification

Relative abundances of *S. oneidensis* MR-1 occurring in the top, middle and bottom two-inch sections of the inoculated and uninoculated columns were measured by qPCR after the completion of column operation. Detection of *S. oneidensis* MR-1 was confirmed at all depths in the inoculated column at an average density across the three layers of 3.9 ± 0.89 × 10^2^ CFU/mL. *S. oneidensis* MR-1 was not detected at any depth in the uninoculated column. These results indicate that the process used to inoculate *S. oneidensis* MR-1 on the anthracite media was successful and supports the hypothesis that the presence of these Mn-reducing bacteria contributed to the additional observed Mn-release. To the authors’ knowledge, no prior study has specifically confirmed the presence of *S. oneidensis* specifically on WTP media, though other species of Mn reducers have been identified (Cerrato et al., 2010). In this specific study, *S. oneidensis* MR-1 was selected as a well-known and highly characterized model, with well-defined gene targets for monitoring, and thus served well in its purpose of representing conditions with confirmed Mn-reducers inoculated.

Relative abundances of *mtrB* genes in the top, middle and bottom two-inch sections of the inoculated and uninoculated columns were also measured by qPCR. Results indicated that the *S. oneidensis* MR-1 inoculated columns contained more *mtrB* gene copies. The average Ct value for the *S. oneidensis* MR-1 inoculated columns was 27 ± 0.26 compared to uninoculated column, which had an average C_t_ value of 38 ± 0.21 (where C_t_ values are inversely proportional to target gene copy numbers). These findings further support the conclusion that there were far more microorganisms that had the ability to reduce Mn in the inoculated column.

#### 3.2.2 Potential effects of aging on the bioavailability of Mn in MnO_x(s)_ coatings

**Fig. 3:**
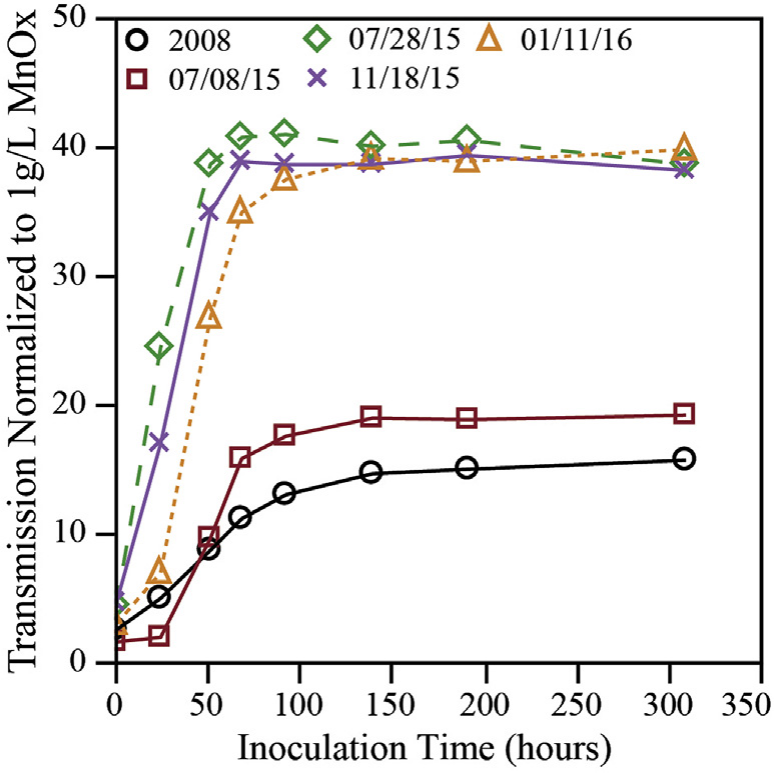
Change in transmission at 540 nm as a measure of extent of Mn reduction that occurred with time in vials containing MnO_x(s)_ samples and uniformly inoculated with *S. oneidensis* MR-1. Each plot represents a distinct MnO_x(s)_ solid sample, named for the date it was collected or generated, initially added at 0.70 g/L for 07/28/15, 01/11/16, and 11/18/15 samples and 0.35 g/L for 2008 and 07/08/15 sample to achieve an initial transmission of 4–8%. Values plotted are averages of triplicate vial incubations, normalized to transmittance measured for 1 g/L of the corresponding MnO_x(s)_.

Fig. 3 presents normalized transmittance values, equivalent to a 1 g/L MnO_x(s)_ agar vial. Normalization of transmittance measurements to that of a 1 g/L MnO_x(s)_ agar vial enabled relative comparisons of Mn reduction capacities. Notably, the 2008 and 7-8-15 solids were characterized by the least extent of Mn reduction over the duration of the study. All the other MnO_x(s)_ solids evaluated in this study were comparable in the observed extent of Mn reduction and corresponding increase in sample percent transmittance.

**Fig. 4:**
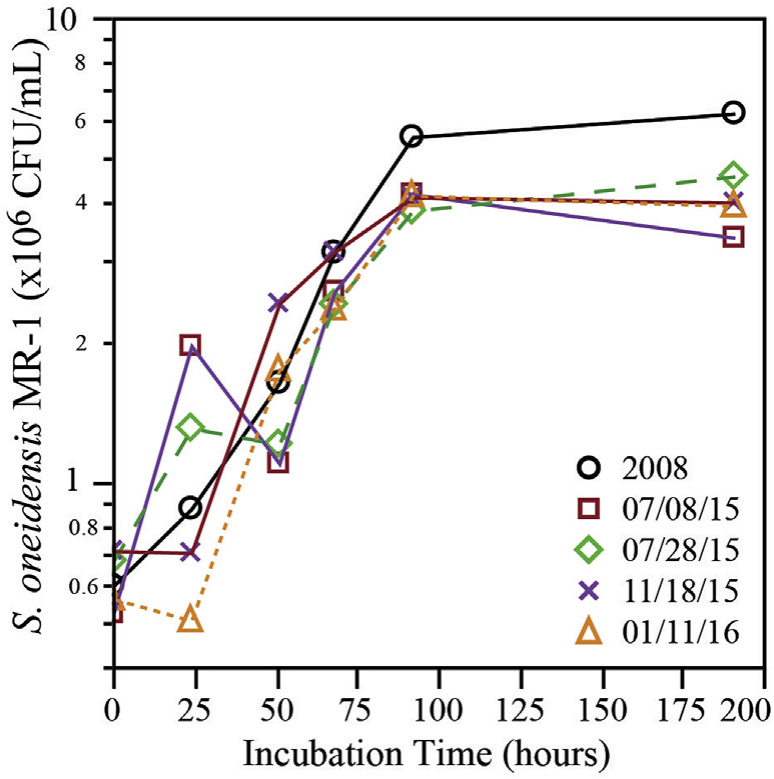
Average *S. oneidensis* MR-1 counts (CFU/mL equivalents) of triplicate vials incubating different MnO_x(s)_ samples (indicated by the dates in the legend) measured via qPCR.

Average *S. oneidensis* MR-1 cell counts were compared among triplicate vials via qPCR over the course of the MnO_x(s)_ incubations (Fig. 4). An ANOVA test confirmed that mean cell concentrations were highly similar across all the MnO_x(s)_ agar vials throughout the experiment (F < F_crit_; P < 0.05). Nonetheless, differences in Mn reduction among the vials were clearly observed (Fig. 3), indicating that variances in *S. oneidensis* MR-1 populations between vials were not responsible for the differences. Further, for the 2008 and 7-8-15 MnO_x(s)_ samples, even though the *S. oneidensis* MR-1 count increased over time, Mn reduction rates were slower than observed for the other samples. The 7-28-15, 11-18-15 and 1-11-16 MnO_x(s)_ samples had the same density of *S. oneidensis* MR-1, but could reduce more MnO_x(s)_. Because the 2008 and 7-8-15 vials had similar *S. oneidensis* MR-1 counts, but lower extent of observed Mn reduction, this pointed to a reduced bioavailability of the MnO_x(s)_ present in these two more “aged” samples.

The ability of microbes to reduce freshly precipitated MnO_x(s)_ when coupled with the oxidation of various organic compounds has been reported by a wide variety of authors, with one of the seminal papers being published by Lovley and Phillips (1988). Aging has been shown to produce chemical and structural changes in the forms of MnO_x(s)_ present (Cui et al., 2010; Hinkle et al., 2016). Likewise, variations in MnO_x(s)_ crystalline structure affect Mn reactivity (Stone, 1987). Burdige et al. (1992) showed that *S. oneidensis*MR-1 could reduce amorphous (more recently precipitated) forms of MnO_x(s)_ (δ-MnO_2(s)_and birnessite) much more readily than occurred when trying to reduce highly-crystalline pyrolusite (MnO_2(s)_).

It is possible to speculate how MnO_x(s)_ aging and potential reduced bioavailability to Mn reducing bacteria could be related to observed Mn release occurring in WTPs with MnO_x(s)_-coated media. Typical anthracite or sand filtration media have a long service life (often well over ten years) due to media durability (Edzwald, 2011). In WTPs employing free chlorine, addition to the filter promotes soluble Mn removal (leading to MnO_x(s)_ deposition on the media) and results in a significant amount of MnO_x(s)_ accumulation over years. Tobiason et al. (2008) showed that MnO_x(s)_ coatings on anthracite media from surface WTPs addressing seasonal elevated Mn concentration issues often had a “tree-ring-like” structures, with many bands of high MnO_x(s)_ placed throughout the coating. Such deposition over many years would provide opportunity for chemical aging and structural changes in the MnO_x(s)_ present that could decrease Mn bioavailability from the coatings. This would correspond with the observation (Knocke, published data) that significant residual MnO_x(s)_ coating may be present on filter media following an observed Mn release event from the filter. The presumption would be that only the more recently deposited MnO_x(s)_ coating was available for microbial reduction due to its less crystalline structure.

## 4 Conclusions

Bench-scale filter column studies served to demonstrate the potential of Mn-reducing microorganisms (specifically *S. oneidensis* MR-1) to contribute to the Mn-desorption phenomenon occasionally seen in WTPs when pre-filtration free chlorine is removed. Specific conclusions that can be derived from this study include:

• Inoculation of column media with the Mn-reducer *S. oneidensis* MR-1 was associated with increased rates of Mn desorption.
• *S. oneidensis* MR-1 residing on the MnO_x(s)_ surface was able to remain viable and reduce Mn after sustained (up to five days) contact with free chlorine.
• MnO_x(s)_ age and crystalline structure could play an important role in the bioavailability of Mn to Mn-reducing organisms.

## Declaration of interests

☒ The authors declare that they have no known competing financial interests or personal relationships that could have appeared to influence the work reported in this paper.

☐The authors declare the following financial interests/personal relationships which may be considered as potential competing interests:

## Appendix A Supplementary data

Mn SI Mn SI
