# Supplementary material for: Interference of manganese removal by biologically-mediated reductive release of manganese from MnOx(s) coated filtration media
Source: Water Res X. 2018 Nov 13;1:100009. doi: 10.1016/j.wroa.2018.100009 (PMC6549940; doi:10.1016/j.wroa.2018.100009)
Supplement: Mn SI [file mmc1.docx]

**Supplementary Information**

**Title:** Interference of Manganese Removal by Biologically-Mediated Reductive Release of Manganese from MnO_x(s)_ Coated Filtration Media

**Author names and affiliations:**

Lindsay E. Swain a, William R. Knocke a, Joseph O. Falkinham III b, Amy Pruden a,*

a VT Via Department of Civil and Environmental Engineering, Virginia Polytechnic Institute and State University, Blacksburg, VA 24061, USA

b VT Biological Sciences Department, Virginia Polytechnic Institute and State University, Blacksburg, VA 24061, USA

*** Corresponding author:** Amy Pruden, apruden@vt.edu

**Present/permanent address:** Via Department of Civil and Environmental Engineering, Virginia Tech, Blacksburg, VA 24061

Table S1. Descriptions of Selected Protein Sequences for MtrB MUSCLE Alignment

| Location | Description | Genus and Species |
| --- | --- | --- |
| NC-014318.1 | Two-component system histidine kinase | Amycolatopsis mediterranei U32 |
| NC-014318.1_1 | Two-component system histidine kinase | Amycolatopsis mediterranei U32 |
| NC-014318.1_2 | Two-component system histidine kinase | Amycolatopsis mediterranei U32 |
| NC-014318.1_3 | Two-component system histidine kinase | Amycolatopsis mediterranei U32 |
| NC-014318.1_4 | Two-component system histidine kinase | Amycolatopsis mediterranei U32 |
| NC-008611.1 | Two-component sensory transduction histidine kinase MtrB | *Mycobacterium ulcerans* Agy99 |
| NC-004347.2 | Extracellular iron oxide respiratory system outer membrane component MtrB | Shewanella oneidensis MR-1 |
| NC-002945.3 | Two component sensory transduction histidine kinase MtrB | Mycobacterium bovis AF2122/97 |
| NC-002677.1 | Two-component system sensor kinase | Mycobacterium leprae TN |
| NC-000962.3 | Two component sensory histidine kinase MtrB | Mycobacterium tuberculosis H37Rv |
